# Evaluation of the Context-Based Prospective Memory (CBPM) task in neurotypical middle-aged and older adults: psychometric properties and sensitivity to age-related cognitive changes

**DOI:** 10.1590/2317-1782/e20250176en

**Published:** 2026-04-17

**Authors:** Siri Vikram, Sharon Ashley, Gagan Bajaj, Dasmine Fraclita D’Souza

**Affiliations:** 1 Department of Audiology and Speech Language Pathology, Kasturba Medical College Mangalore, Manipal Academy of Higher Education, Manipal, India.

**Keywords:** Prospective Memory, Social Context, Psychometric Properties, Healthy Aging, Cognitive Assessment

## Abstract

**Purpose:**

Prospective Memory (PM) is the ability to remember intentions for future execution which plays an important role in communication and is vulnerable to age-related decline. Context-Based Prospective Memory (CBPM) task, a novel test in Indian scenario evaluated psychometric properties among young adults. This study aimed to evaluate the psychometric properties of CBPM task among the neurotypical middle-aged and older adults and to investigate its sensitivity to age-related changes.

**Methods:**

A total of 80 participants (40 middle-aged, 40 older adults) completed the CBPM task and the Memory for Intentions Screening Test (MIST). Psychometric properties like internal consistency, test-retest reliability and convergent validity were evaluated. Age-related differences were examined using previously established CBPM task data from young adults.

**Results:**

CBPM task demonstrated strong internal consistency (Cronbach’s α= 0.875 for middle-aged and 0.715 for older adults), Excellent test-retest reliability for total performance scores (ICC=0.93 and 0.81) for middle-aged and older adults respectively and error classification (ICC= 0.97 and 0.99). Similarly inter-rater reliability also indicated strong reliability for the total PM performance (ICC=0.91 and 0.85), and error classification (ICC=0.97 and 0.99). Significant convergent validity was also observed with MIST, suggesting that both the tasks perform similarly. On assessing the tool’s sensitivity to age-related changes in PM abilities, significant declines were observed in middle-aged and older adults compared to young adults affirming the age-related cognitive deterioration.

**Conclusion:**

The CBPM task is an effective and reliable tool to measure PM across age groups, demonstrating strong psychometric properties and sensitivity to identify the age-related decline.

## INTRODUCTION

Prospective memory (PM), or the capacity to recall and achieve the aimed task at the right time^(1)^, is a cornerstone of daily life. Whether it’s delivering a message, making a scheduled phone call, or remembering to take medication, PM underpins our ability to navigate personal, social, and professional responsibilities. Among aging adults^(2)^, PM has a particularly pivotal role in preserving independence and ensuring wellness across various domains^(3)^. Unlike retrospective memory, which involves recalling past events or information, PM is characterized by self-initiated retrieval processes, where one must independently act on a prior intention when prompted by a relevant cue, rather than external demands to remember^(4)^. From attending appointments to paying bills, PM tasks are deeply woven into the fabric of everyday functioning, underscoring their vital importance in preserving an individual’s welfare and independence^(5)^.

Beyond its role in everyday tasks, PM is also fundamental to effective communication^(6)^. It enables crucial activities like recalling factual details in conversations, adhering to response timelines through email, initiating or answering telephone calls promptly or re-dialing without delay, being available for crucial meetings or planned commitments, and relaying critical messages accurately^(6)^. PM skills are also needed to fit into social norms, i.e., greeting others, making eye contact, and engaging appropriately in dialogues thus making communication more effective and stronger. PM's contribution to communication is indispensable in meeting social expectations and fostering meaningful relationships, both personal and professional. Communication related failure in PM can result in misunderstandings. Compared to retrospective memory, PM carries a moral weight; failures are often perceived as signs of unreliability, potentially damaging an individual’s credibility and self-esteem. This highlights the profound social importance of PM in maintaining trust and meaningful interactions^(6)^.

PM is particularly sensitive to age-related cognitive decline, and several studies have demonstrated that cognitive performance deteriorates with age^(7)^. These deficits are not only evident in healthy aging, but also well documented in cognitive-communication conditions, like mild cognitive impairment (MCI), dementia, and traumatic brain injury (TBI)^(8)^. Given its functional relevance, PM has increasingly been advocated as an essential component in cognitive-communication assessments much like other cognitive zones such as working memory, short-term memory, and executive function.

Neuroanatomically, age-related declines in PM performance have been connected to structural and functional modifications in key brain regions responsible for the formation, retention, initiation and execution of intentions^(9)^.Two distinct frontoparietal networks are considered critical for PM. The dorsal frontoparietal network is primarily involved in the formation and retention of intention by allowing the individual to keep the future tasks in mind while engaging in ongoing activities, primarily through strategic monitoring mechanisms^(10)^. While ventral frontoparietal network facilitates the initiation and execution of intentions by enabling spontaneous retrieval of intended actions in response to relevant internal or external cues^(10)^.Another key region implicated in PM is the anterior prefrontal cortex (aPFC), which plays an important role in PM function. Age-related structural decline in the aPFC has been shown to negatively impact PM functioning^(10)^. These neuroanatomical changes help explain the observed behavioural impairments in PM among older individuals and highlight the need for assessment tools sensitive to such age-related shifts in brain function.

A wide array of tools is currently utilized to assess and measure PM, including experimental tasks, single-trial approaches and self-report questionnaires as well as paper-and-pencil test batteries^(11)^. Test batteries include the Rivermead Behavioural Memory Test (RBMT)^(12)^, the Cambridge Test of Prospective Memory (CAMPROMPT)^(13)^, the Memory for Intentions Screening Test (MIST)^(8)^, and the Royal Prince Alfred Prospective Memory Test (RPA-Pro-Mem)^(14)^. Single-execution protocols include the Envelope task^(15)^, the Prompt card task, the Telephone test^(11)^, and the Key test^(16)^. Questionnaires include the Prospective Memory Questionnaire (PMQ)^(17)^, the Prospective and Retrospective Memory Questionnaire (PRMQ)^(18)^, the Comprehensive Assessment of Prospective Memory Questionnaire (CAPM)^(19)^, and the Brief Assessment of Prospective Memory Questionnaire (BAPM)^(20)^. Analytical and investigative paradigms consist of the^(21)^, the Test Écologique de Mémoire Prospective (TEMP)^(22)^, the Virtual Week, and Actual Week^(23)^.

Despite the variety of tools available to assess PM, traditional test batteries like RBMT and MIST, often lack the ecological validity as they are administered in controlled environments that do not accurately reflect real-world cognitive demands. In everyday life, PM tasks are associated with specific environmental and situational cues. Since contextual relevance is most effective when aligned with cultural norms, incorporating culturally appropriate contexts during PM assessment may reduce cognitive load and improve PM performance^(24)^. Despite the fact that PM capacities are an essential component in many cognitive testing batteries, there exists a notable lack of culturally relevant, context-based PM tests with adequate reliability and validity in India.

Acknowledging these limitations, a novel context-based PM task (CBPM)^(25)^ was developed to provide a culturally relevant and ecologically valid tool for assessing PM in an Indian context, building on the framework established by MIST. It was validated among young adults in accordance with the assessment parameters outlined in MIST. This innovative task incorporates a virtual shopping mall environment, simulating real-world scenarios to evaluate PM abilities effectively. It comprises both an ongoing activity and a PM component, designed to mirror everyday cognitive demands. Its psychometric properties are being systematically evaluated through a multiphase approach. The first phase involved the development of the task and its initial validation in a young adult population^(25)^. To further establish CBPM task as a robust assessment tool, it is crucial to evaluate its psychometric properties in middle-aged and older adults, as well as to examine its sensitivity to age-related cognitive changes, prior to its application in clinical populations or individuals with cognitive-communication disorders. Hence, as a second phase, the current study aims to establish the psychometric properties of the CBPM task, specifically assessing its internal consistency, test-retest reliability, inter-rater reliability and convergent validity among typically aging middle-aged adults, old-aged adults, and to determine its utility in detecting age-related changes in PM.

## METHOD

The research study employed cross-sectional methodology, and the Institutional Ethics Committee granted its ethical approval (IEC KMC MLR 04/2024/240). This study tracked the Strengthening the Reporting of Observational Studies in Epidemiology (STROBE) parameters to warrant clarity as well as completeness in reporting^(26)^. Informed consent and signatures were obtained from all eligible participants prior to their participation.

### Participants

A total of 80 participants [40 middle-aged adults (Male: N - 6, Mean age - 48.3, Standard deviation (SD) - 7.15, Female: N - 34, Mean Age - 53.3, SD - 6.21) and 40 old aged adults (Male: N - 11, Mean age - 73.2, SD - 5.04, Female: N - 29, Mean Age - 72, SD - 5.88)] were recruited from geriatric clinics, old-age homes and our institute's outpatient department. Participants with a minimum qualification of 12th standard /2nd PUC with a high proficiency in English (>7 score) on the Language Experience and Proficiency Questionnaire (LEAP-Q)^(27)^. Socioeconomic status was determined using the Modified Kuppuswamy’s Socioeconomic Status Scale^(28)^, and only individuals from a middle socioeconomic background were included. Only individuals with normal or corrected vision and hearing were considered for inclusion; those exhibiting current or past psychological or neurological conditions were not eligible as measured through WHO Ten Questions screen test^(29)^.

## Materials

### The context-based PM task

**Figure 1:**
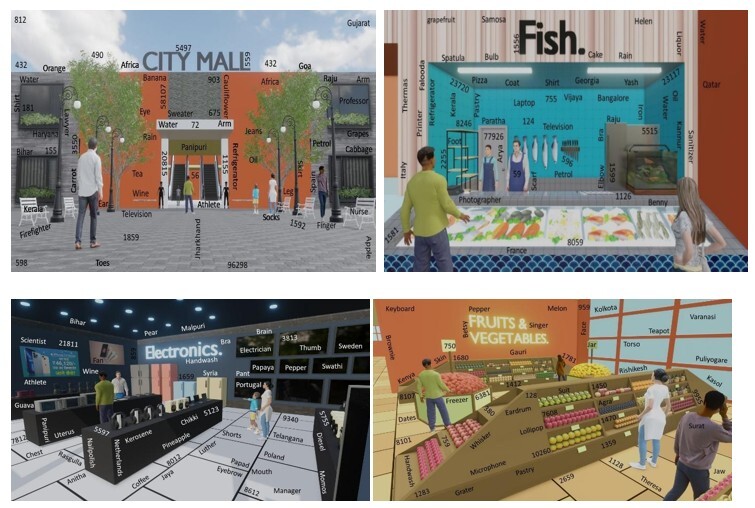
Rendered images of different sections from the task as adapted from D’Souza et al.^(25)^

The CBPM task developed by D’Souza et al.^(25)^ includes event- and time-driven PM trials, together alongside an ongoing task incorporated within the environment of a shopping mall. A total of 40 images representing 20 distinct locations — including retail stores (cosmetics, clothing, cutlery, electronics, toys, footwear), food outlets (bakery, fish shop, meat shop, cold storage, chocolate shop, food court), and other functional areas (baggage counter, travel store, home décor, billing and customer service counters, entrance, exit, and parking lot) — are used to create a partially immersive experience. These images are embedded with ongoing PM targets, as shown in some of the sample depictions in Figure 1.

The ongoing task requires the participants to look out for specific digits or words embedded within various images of a shopping mall. Participants are instructed to identify numbers containing the combination ‘81’ (e.g., 7814, 581) within each scene. To increase task complexity, words are also embedded alongside the numbers to serve as distractors.

The PM task is non-focal in essence, requiring individuals to perform a designated action, either verbally or nonverbally, at a specific instant or place within the mall. A total of ten PM tasks are administered, categorized across three dichotomies: the cue of the stimuli (event-driven or time-driven, i.e., implementing an aimed activity at a pre-established time or place within the mall), response format (action or spoken, i.e., performing a nonverbal or verbal intention), and the length of the delay (short or lengthy duration, i.e., less than or more than 4 minutes between the delivery of the intention and its performance). The cognitive load is maintained between 1 and 5 PM targets. Each PM trial is described along with its presentation sequence, associated cognitive load, and classification under the three dichotomies, as depicted in Table 1.

**Table 1:** PM trials, the presentation sequel, and the corresponding cognitive load

Trails	Instructions	Cue	Response modality	Time duration	Order of execution	Cognitive load
**1**	“When you come across seafood, count and tell the me the number of salesmen present.”	Event-based	Verbal	Short	1	4
**2**	“Ask me if I need anything from the mall,10 minutes from now”	Time-based	Verbal	Long	4	5
**3**	“When you are at the section where you can buy lollipops, point out any object that is green in colour”	Event-based	Action	Short	2	5
**4**	“After a duration of 11 minutes from now, let me know the name of the store you are situated in”	Time-based	Verbal	Long	6	4
**5**	“Write the word ‘GOLDFISH’ when you reach the section where you see T shirts”	Event-based	Action	Long	5	4
**6**	“In the next 3 minutes, write down your phone number on the sheet/notepad next to you”	Time-based	Action	Short	3	5
**7**	“When you reach the section where you can order pizza, remember to show the me ‘J’ key on the keyboard”	Event-based	Action	Long	8	3
**8**	“After 8 minutes from now, show me a thumbs up”	Time-based	Action	Long	9	2
**9**	“In 2 minutes from now, name 3 items you can see in the room”	Time-based	Verbal	Short	7	4
**10**	“Tell the name of your bank, when you see a white car”	Event-based	Verbal	Short	10	1

Note. Adapted from the earlier version of the CBPM task, used in a study among young adults^(25)^, with a balanced representation of PM tasks across dichotomies

The administration of CBPM task, in the present research, followed the same protocol outlined in D’Souza et al^(25)^, with a practice trial done prior to the main task. Participants received the following instructions:

*You will be shown various scenes from a mall, each containing random numbers and words. Your task is to identify numbers that include the combination '81' (for example, 7814, 481, 812). While doing this, I will assign you additional tasks that you must remember and perform at the correct time, without any reminders. A digital clock is provided to help you track time for these tasks*.

The investigator recorded the outcome of each of the subjects on both the ongoing tasks and PM trials, noting any errors made.

The scoring criteria for the CBPM task, in the present work, is identical to that defined in D’Souza et al.^(25)^. Accuracy for PM targets was assessed on a scale of ‘0,’ ‘1,’ or ‘2,’ depending on task type. For time-based tasks, a timely execution was scored as ‘2,’ while a response with a slight timing error (within ±30 seconds) was scored as ‘1,’ and missed responses received a score of ‘0.’ For event-based tasks, accurate execution was scored as ‘2,’ while missed responses were marked as ‘0.’ Six subscale measurements were calculated with respect to the predefined dichotomies: short-duration tasks, long-duration tasks, time-based tasks, event-based tasks, action responses, and verbal responses, and a total overall score of PM was derived by summing the scores of subscales with a maximum possible score of 60.

Additionally, the type of errors displayed by participants were documented and classified based on the MIST error taxonomy. The first type of error, termed Prospective Memory Failure (PF), was recorded when a member was completely unsuccessful to respond to a PM cue. The second error type, known as a Task Substitution (TS) error, was noted when the participant performed an action suitably but executed a task intended for a different cue. The third error, Loss of Content (LC), was assigned when the participant recognized the PM cue but was unable to recall part or all of the intended action. The fourth, Loss of Time (LT) error, was marked when the correct task was performed, but at an incorrect time point. Finally, a Random Error (RE) was coded for any responses that did not fit into the previous error categories. Each error type is associated with a specific score.

Finally, performance of the ongoing task was evaluated independently, where each correct detection of the target (‘81’) was awarded a score of ‘1,’ yielding a maximum possible score of 138. PM performance, error characteristics, and ongoing task outcomes were collectively used to assess the participant's cognitive functioning within the CBPM task.

### MIST

Consistent with the protocol followed by D’Souza et al.^(25)^ MIST was employed in the present study to establish the convergent validity of CBPM task in middle aged and older adults. MIST was developed by Raskin^(8)^ and is considered a standardized measure to assess PM. It comprises time-based and event-based PM tasks embedded within an ongoing word search activity, requiring participants to remember and execute specific intentions at designated times or in response to specific cues. It demonstrates good psychometric properties, with established internal consistency, inter-rater reliability, and construct validity, and has been shown to effectively differentiate between healthy adults and clinical populations with memory impairments.

### Procedure

The stimulus of the shopping mall scenes was presented through a LENOVO IdeaPad 3 with a 15-inch display. The test was conducted in a quiet environment. Each participant had to complete two tasks: the CBPM task and the MIST. The sequence of task administration was counterbalanced, with one half of participants beginning with the CBPM task and the other half with the MIST, ensuring consistency across both the study groups. The overall time taken for the sessions was approximately 45 minutes, with the CBPM task taking 25 minutes and MIST around 20 minutes. A digital clock was positioned in front of each participant to aid in time management for time-based tasks, while pen and paper were provided for tasks necessitating written responses. The examiner delivered instructions verbally, and participants' responses were recorded in real time.

### Data analysis

A statistical analysis was done using the Jamovi software (2.3.28) and IBM SPSS (version 29). Mean and Standard deviation were obtained through descriptive statistics for each subscale of MIST and the CBPM task. The reliability of the subscales was assessed by examining internal consistency using both Cronbach’s alpha and Spearman-Brown’s split-half reliability analysis. Additionally, Spearman’s correlation was applied to examine the connection between individual trials and subscales of the CBPM task. Convergent validity was assessed across middle- aged adults and older adults by comparing their performance on CBPM task and MIST scores using McNemar’s test. To further support convergent validity, an error code correlation analysis was conducted between the MIST and CBPM task using Spearman’s correlation coefficient. The reliability of the PM scores from the CBPM task were evaluated using both test-retest and inter-rater reliability measures for the total performance scores as well as error scores. These reliability metrics were quantified using intraclass correlation coefficients (ICCs).

To evaluate the utility of the CBPM task in detecting age-related changes in PM, data from young adults reported by D’Souza et al.^(25)^ were compared with middle-aged and older adult data obtained in the present study. Shapiro-Wilk’s test was done to examine the normality of the data. Further, Kruskal-Wallis’s test was done to study the age-linked effects between the three groups. Post hoc analysis using Dwass-Steel-Critchlow-Fligner was done to identify the differences between the different age groups. Additionally, ongoing task accuracy was analysed using one-way ANOVA, followed by Games-Howell post hoc comparisons.

## RESULT

### Psychometric properties of CBPM task

Descriptive performance of the middle-aged and older adults on CBPM task across scales and the total performance scores has been provided in Table 2. Among the middle-aged adults, the short-duration task (M=6.13), time-based task (M=5.13) and the verbal response task (M=5.15) were observed to have the highest scores, while the long-duration task (M=3.98), event-based task (M=4.97) and the action response task (M=4.95) showed comparatively lower scores. A similar pattern was seen in older adults, where performance of short-duration task (M= 7.55), time-based task (M= 6.67) and verbal response task (M= 6.72) was relatively better than long-duration task (M= 3.15), event-based task (M=4.03) and action response task (M=3.98).

**Table 2:** Mean, median, and standard deviation of the subscales wise and total performance on CBPM task of middle-aged and older adults’ groups

Subscale	Max	Mean		Median		SD	
		MA	OA	MA	OA	MA	OA
**Short-duration task**	10	6.13	7.55	6	8	2.10	1.28
**Long-duration task**	10	3.98	3.15	4	3	2.14	1.27
**Time-based task**	10	5.13	6.67	5	7	1.60	1.1
**Event-based task**	10	4.97	4.03	5	4	2.58	1.17
**Verbal-response task**	10	5.15	6.72	5	7	1.85	1.4
**Action-response task**	10	4.95	3.98	5	4	2.19	1.31
**Total score**	60	30.3	32.1	30	33	9.89	3.2
**Ongoing Accuracy**	138	95.8	86.1	94	87	17.3	9.93

MA-Middle Age; OA-Old Age; SD-Standard Deviation

The percentage distribution of error types in the CBPM task across middle-aged and older adults is presented in Tables 3 and 4. Among the error types, PF and LC were the most frequent across both age groups, though PF was notably higher in older adults. LT errors were also very evident in older adults as compared to middle-aged adults. TS, PL, and RE were relatively infrequent in both groups. Overall, the error distribution reflects age-related trends in PM performance, with older adults exhibiting more cue detection and timing difficulties compared to middle-aged adults.

**Table 3:** Percentage of occurrence of each error type in the PM trials of the context-based PM task in middle-aged adults

TRIALS	NE	LC	LT	PF	TS	RE	PL
**1**	16	7	2	12	2	0	1
**2**	11	10	0	11	1	0	7
**3**	24	5	6	3	2	0	0
**4**	11	4	4	21	0	0	0
**5**	12	11	1	13	2	0	1
**6**	3	4	3	29	1	0	0
**7**	29	2	3	5	1	0	0
**8**	29	5	0	2	2	0	2
**9**	17	3	5	13	2	0	0
**10**	28	5	0	5	2	0	0

NE- No Error; PF-Prospective memory failure; TS-Task substitution; LC-Loss of content; LT-Loss of time; RE- Random error; PL-Place Losing

**Table 4:** Percentage of occurrence of each error type in the PM trials of the context-based PM task in old age adults

TRIALS	NE	LC	LT	PF	TS	RE	PL
1	27	11	0	2	0	0	0
2	14	9	0	14	0	0	3
3	34	2	2	2	0	0	0
4	18	8	5	9	0	0	0
5	2	16	0	21	0	1	0
6	2	7	6	25	0	0	0
7	40	0	0	0	0	0	0
8	2	17	1	20	0	0	0
9	17	4	13	6	0	0	0
10	35	0	3	1	0	1	0

NE- No Error; PF-Prospective memory failure; TS-Task substitution; LC-Loss of content; LT-Loss of time; RE- Random error; PL-Place Losing

### Consistency and Reliability

To determine the internal consistency, analyses were conducted using Cronbach’s alpha and the Spearman-Brown split-half method. The analysis demonstrated a robust internal stability among the six subscales, as reflected by a Cronbach’s alpha coefficient of 0.875 for middle-aged adults and 0.715 for older adults. Furthermore, the Spearman-Brown coefficient was found to be 0.784 and 0.952 for middle-aged and older aged adults respectively, indicating a good split-half reliability.

Spearman’s correlation analysis examined the relationship between individual trial performance and the corresponding subscale scores. The results, as presented in Table 5, revealed a statistically significant correlation in most cases, with a few exceptions (action response in trial 3, time cue in trial 7, and event cue in trial 10).

**Table 5:** Spearman’s rank correlation between the performance on each trial and the subscale scores

Trials	SD	LD	TB	EB	V	A	PMT
**1**	0.645*	-0.104	0.051	0.506*	0.539*	-0.039	0.447*
**2**	0.494*	0.039	0.016	0.496*	-0.011	0.567*	0.431*
**3**	0.461*	-0.108	0.426*	-0.035	0.199	0.128	0.284*
**4**	0.123	0.400*	0.590*	0.035	0.591*	-0.059	0.410*
**5**	-0.055	0.294*	-0.174	0.335*	-0.119	0.372*	0.190
**6**	-0.027	0.263*	0.329*	-0.022	0.360*	-0.125	0.157
**7**	0.430*	-0.262	0.189	0.073	0.290*	-0.110	0.188
**8**	-0.299	0.453*	-0.341	0.372*	-0.347	0.486*	0.042
**9**	-0.070	0.534*	0.442^*^	-0.028	-0.023	0.447*	0.310
**10**	0.295*	0.049	0.210	0.213	0.572*	-0.196	0.288*

* p<0.05

### Test-Retest Reliability and Inter-Rater Reliability

To assess the reliability, 20% of the total sample (n=8 middle-aged adults; n=8 older adults) was selected. Test–retest reliability was assessed by re-administering the task after a two-week interval, while inter-rater reliability was determined based on independent ratings provided by two trained assessors.

Test–retest reliability was examined using intraclass correlation coefficients (ICCs). For the total performance scores, ICCs were 0.931 [95% CI: 0.69–0.98; F(7, 7.1) = 24.8, p < 0.001] in middle-aged adults and 0.813 [95% CI: 0.36–0.95; F(7, 7.5) = 10.8, p < 0.005] in older adults, indicating good test–retest reliability. For error classification, ICCs were 0.977 [95% CI: 0.98–0.99; F(6, 7) = 692, p < 0.001] in middle-aged adults and 0.997 [95% CI: 0.98–0.99; F(6, 7) = 573, p < 0.001] in older adults, reflecting excellent test–retest reliability.

Inter-rater reliability for total performance scores yielded ICCs of 0.917 [95% CI: 0.67–0.98; F(7, 8) = 23.1, p < 0.001] for middle-aged adults and 0.846 [95% CI: 0.45–0.96; F(7, 7.9) = 12, p < 0.001] for older adults, indicating strong inter-rater reliability. For error classification, ICCs were 0.977 [95% CI: 0.88–0.99; F(6, 7) = 84.9, p < 0.001] and 0.997 [95% CI: 0.98–0.99; F(6, 7) = 505, p < 0.001] in middle-aged and older adults, respectively, demonstrating excellent inter-rater reliability.

### Convergent validity

To assess convergent validity, participants' performance on the CBPM task was compared with their scores on MIST. McNemar’s test was conducted to evaluate the alignment of the two measures in assessing PM abilities. To distinguish between high and low performance on each task, scores were categorized into two groups based on the median values for both tasks. The results of the test indicated no statistically significant difference between the proportion of high and low performers across MIST and CBPM task scores in the middle-aged individuals (χ^2^=0.6, p=0.439) and old -aged adults (χ^2^=2.33, p=0.127), thus supporting convergent validity.

Additionally, Spearman’s correlation investigation was conducted to examine the relationship among various categories of errors made by participants in the MIST and CBPM tasks across the two groups. The results showed a high positive correlation between the totals of different errors in both tasks, with the strongest correlation observed in the older adult group (ρ = 0.937, p = 0.002), followed by middle-aged adults (ρ = 0.919, p = 0.003).

### Utility of CBPM task in assessing age-related changes in PM abilities

To evaluate age-related changes in PM, the total performance scores of CBPM task from young adults^(25)^ were compared with the total performance scores of middle-aged and older adults in the present study, with Shapiro-Wilk tests indicating non-normality across all groups (p < 0.001).

**Figure 2:**
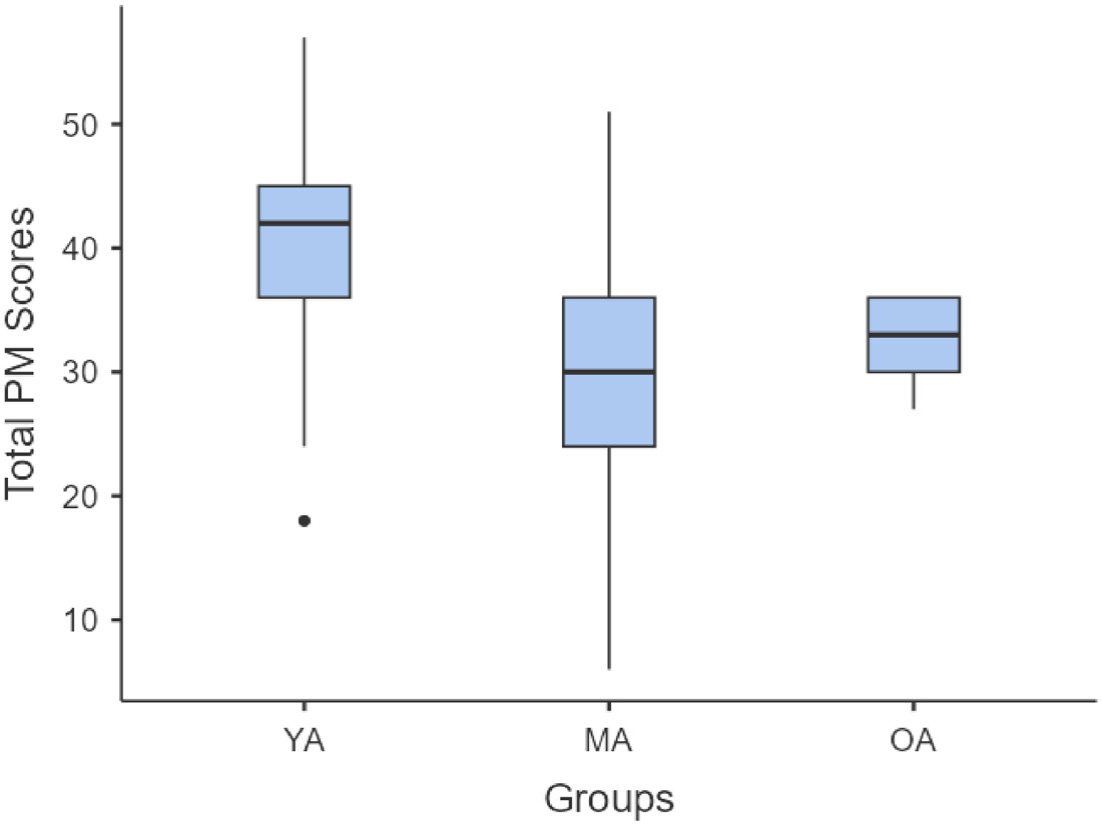
Box plot depicting the distribution of the total PM scores across young adults, middle-aged adults and older adults

A Kruskal-Wallis test revealed a statistically difference in PM performance among the three ages (χ^2^ = 30.7, p < 0.001). Post hoc analysis using the Dwass-Steel-Critchlow-Fligner test showed that young-adults performed significantly better than middle-aged (W = 6.17, p < .001) and older-aged adults (W = 7.02, p < 0.001), however, no significant difference was found between middle-aged and older adults (p = 0.28) (Figure 2).

**Figure 3:**
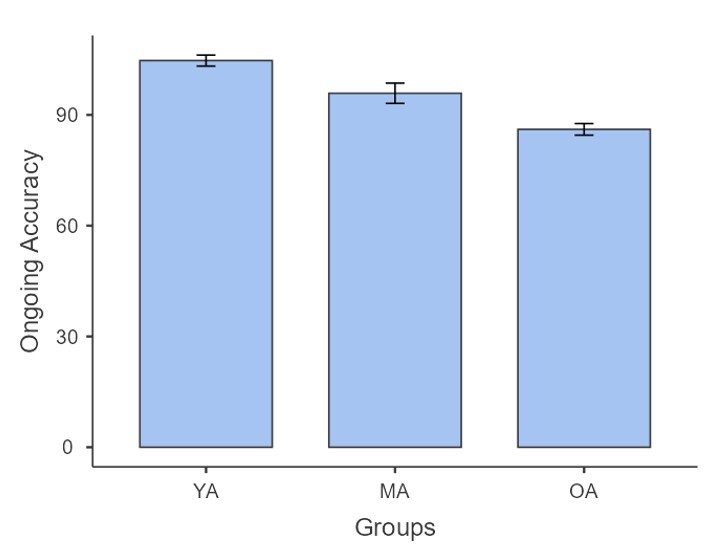
Bar graph depicting the distribution of the ongoing accuracy scores across young adults, middle-aged adults and older adults

For the ongoing task scores, the Shapiro-Wilk test indicated normal distribution across groups. Welch’s one-way ANOVA was performed indicating a notable distinction among the groups, F (2, 78) = 36.8, p < .001. Games-Howell post hoc comparisons indicated that young adults outperformed both middle-aged adults (M = 8.85, p = 0.017) and older adults (M = 18.63, p < 0.001). Additionally, middle-aged adults scored significantly higher than older adults (M = 9.77, p = 0.008), demonstrating a clear age-related decline in task performance across the three groups (Figure 3).

## DISCUSSION

### Psychometric properties of CBPM task

The CBPM task was developed to address the lack of culturally and contextually relevant PM assessment tools suitable for the Indian population. Existing PM tasks often overlook cultural and environmental factors that influence memory performance. Drawing inspiration from studies like Ouellet^(30)^, which emphasized the effectiveness of virtual shop-based assessments for evaluating memory domains, the CBPM task was designed using a familiar shopping mall scenario featuring diverse locations. Initially validated among young adults^(25)^, the current research aims to establish the psychometric properties of the CBPM task in neurotypical middle-aged adults, older adults and explore its utility in detecting age related PM changes. By a methodical examination of internal consistency, reliability, validity, and group differences in performance, the current research adds to the emerging PM test literature, addressing the lack of contextually relevant PM tasks.

The validation of this measure and the establishment of its psychometric properties in middle-aged and older adults who are healthy cognitively were required to confirm its reliability and broader applicability.

Descriptive performance of CBPM task performance revealed that middle-aged and older adults each demonstrated strengths in different task. Interestingly, the mean total performance score was slightly higher in older adults (M = 32.1) compared to middle-aged adults (M = 30.3). Older adults also seemed to perform better on short duration tasks, time-based tasks and tasks that required verbal responses. This marginal advantage may reflect increased motivation among older adults, potentially driven by heightened awareness of age-related cognitive changes^(31)^. Conversely, middle-aged adults showed superior performance on long-duration, event-based and action-response tasks, which are more dependent on self-initiated retrieval processes.

Assessing the internal consistency and reliability of the CBPM task is essential for determining the effectiveness of the assessment tool^(32)^. In this study, reliability was measured through Spearman-Brown split-half method and Cronbach's alpha among middle-aged and older adults. The CBPM task established high internal consistency and reliability with Cronbach alpha of 0.875 among middle-aged and 0.715 among older adults, confirming that the instrument was effective in the measurement of PM performance among the two groups. The high internal consistency observed across subscales suggests that the test components measure a cohesive construct, making CBPM task a reliable assessment tool. This also corresponds with earlier findings of a strong internal consistency among young adults using CBPM task^(25)^. Additionally, split-half reliability analysis further supports the robustness of the test, indicating that both halves of the assessment produce comparable results, consistent with the findings stated by D’Souza et al.^(25)^. Further, the relationship between individual trial performance and subscale scores was examined through Spearman’s correlation analysis to validate the CBPM task’s ability to measure PM performance consistently. The strong correlations between individuals trial performance and overall subscale scores reinforce the validity of the CBPM task in capturing PM abilities in neurotypical adults of this study. These findings again resonate with the previous research on young adults using this tool^(25)^.

The test-retest reliability results support the stability of the CBPM task over time, reinforcing its utility in assessing PM performance. The high intra-class correlation coefficients observed for total PM performance and error classification indicate strong test-retest between initial and follow-up assessments. This consistency suggests that CBPM task reliably captures individual differences in PM abilities across repeated administrations. Similar conclusions have been drawn in past studies^(8,25)^. Notably, the error classification component exhibited exceptionally high reliability, highlighting its robustness in distinguishing different error types. These findings confirm that the task yields stable and reliable results across repeated administrations, demonstrating good test-retest reliability for both total PM performance and error classification.

Inter-rater reliability for the total PM performance and error classification was high, suggesting consistent ratings across observers. These findings suggest that the rating criteria were well-defined and consistently applied, bolstering the credibility of the observational data. The high reliability across both the total PM performance and error metrics enhances confidence in the validity of the CBPM task and supports the robustness of the results derived from these measures. The strong inter-rater reliability observed in the present study mirrors findings by D’Souza et al.^(25)^ among younger adults, suggesting that the rating procedures are robust across age groups.

Inspired by MIST in terms of its dichotomies, ongoing task component, and established psychometric properties, the CBPM task was developed to provide a comprehensive assessment of PM. Convergent validity analysis further supports its robustness by confirming statistical alignment with these measures. The comparison between CBPM task and MIST scores using McNemar’s test indicated no significant differences across middle-aged adults and older adults, suggesting that both measures assess PM abilities similarly. This alignment, therefore, reinforces the construct validity of CBPM task as a reliable PM assessment tool. Furthermore, Spearman’s correlation analysis revealed a strong positive relationship between error patterns in CBPM task and MIST, with the high correlation observed in older adults, followed by middle-aged adults. The significant correlations across all groups indicate that CBPM task effectively captures PM deficits in a manner consistent with established measures. The earlier study among the young adults using this tool confirms this pattern^(25)^, making it a valid tool among the different age groups.

Error analysis also revealed that LC errors and PF errors were the most frequent errors seen in the two groups. The high occurrence of LC errors may be attributed to the heavy reliance of the content component of PM on retrospective memory^(33)^. Whereas PF errors- representing a complete failure to remember and execute the intended task, can be linked to the overall cognitive deficits, affecting multiple cognitive domains essential for successful PM outcomes^(33)^.

### Utility of CBPM task in assessing age-related changes in PM abilities

The comparative analysis of CBPM task performance across the three groups provides critical insights into the trajectory of PM decline. Comparing the results of the total scores of PM with younger adults, it showed that middle-aged and older adults performed significantly lower than younger adults, reflecting the pronounced age-linked decline in PM abilities. However, the change between middle-aged and older adults was not very significant. This pattern suggests that age-related declines in PM may begin to manifest during midlife and follow a gradual trajectory^(34)^. This decline is consistent with age-related changes in brain regions involved in PM, particularly the aPFC and the frontoparietal networks. These areas are responsible for maintaining and executing future intentions and atrophy of these areas can impair the strategic monitoring and cue detection required for successful PM resulting in poor scores with aging^(10)^.

Similarly, the analysis of ongoing task scores revealed significant distinctions across the three groups. However, when considering ongoing task accuracy, middle-aged adults outperformed older adults (M = 95.8 vs. 86.1) unlike the mean total performance scores, suggesting that while older adults may prioritize the prospective component of the task, their ability to maintain accuracy on the ongoing task may be compromised owing to age-related declines in attention and processing speed^(35,36)^. Age-associated degeneration of cortical areas like posterior parietal cortex and dorsolateral prefrontal cortex can lead to difficulties in dividing and sustaining attention across different tasks^(37)^. This is also seen in the present study where the performance of young adults was found to be higher than that of both middle-aged and older adults indicating a gradual deterioration in cognitive function across adult life. As these brain regions decline with age, the ability to coordinate between ongoing tasks and future intentions also weakens, contributing to poorer PM performance in older adults^(38)^.

In conclusion, the CBPM task developed in this research proves to be a reliable and valid tool for assessing PM abilities comprehensively. Its excellent psychometric properties, as in high reliability, validity, and well-designed error analysis, establish it as a robust assessment measure and the task design incorporated different cognitive loads, retrieval demands, and response formats, allowing for a comprehensive evaluation of PM performance. The integration of an ongoing task, along with PM accuracy evaluation, ensures that the tool closely reflects real-world PM demands. Furthermore, the shopping mall context enhances ecological validity, making the task engaging and relatable for participants. Given its holistic approach and strong methodological foundation, this tool holds significant potential for cognitive-communicative assessments and could be further adapted for diverse populations and clinical applications.

### Limitations and future directions

A key limitation of the current study lies in the generalizability of the CBPM task, which was examined only in neurotypical middle-aged and older adults, as a continuation of earlier validation conducted among young adults. While this is a critical step in establishing normative data and understanding age-related changes in PM performance, the findings do not extend to individuals from diverse socioeconomic and educational backgrounds or to clinical populations. Consequently, the results cannot yet inform the severity of performance impairments in populations with cognitive-communication disorders. Future studies are planned to validate the CBPM task across broader demographic profiles and clinical groups, such as individuals with MCI, to enhance understanding of PM performance and expand the tool’s utility for early detection and intervention. Considering that the CBPM task is currently in the formative stage of development and its construct validity is still being established through a series of studies, methodological details, visual references, and illustrative examples are provided in this manuscript to ensure transparency and support critical evaluation of the task design. Although studies on its psychometric properties in clinical populations are underway to enhance its clinical utility, the CBPM task is presently available for research purposes and can be obtained upon reasonable request from the corresponding author.

## CONCLUSION

This study aimed to extend the validation of CBPM task to middle aged and older adults, establishing strong psychometric properties, including high internal consistency, test-retest reliability and construct validity, as with prior established findings in young adults. The results support that CBPM task is an effective and reliable instrument to measure PM across the adult life span. Its capacity to differentiate age related performance patterns and its strong correlation with established measures like MIST highlight its clinical utility for normative assessment. The utilization of real-world context and varied cognitive demands makes CBPM task representative of functional memory capabilities, and therefore a useful addition to neuropsychological batteries.
